# Factors Influencing Disease Dynamics in Small-Scale Carp Polyculture in Bangladesh

**DOI:** 10.3390/ani14060966

**Published:** 2024-03-20

**Authors:** Partho Pratim Debnath, Pochara Prukbenjakul, Melba G. Bondad-Reantaso, Charles R. Tyler, Channarong Rodkhum

**Affiliations:** 1 Center of Excellence in Fish Infectious Diseases Research Unit (CE FID), Faculty of Veterinary Science, Chulalongkorn University, Bangkok 10330, Thailand; parthoku2004@yahoo.com (P.P.D.);; 2 Department of Veterinary Microbiology, Faculty of Veterinary Science, Chulalongkorn University, Bangkok 10330, Thailand; 3 Fisheries and Aquaculture Division, Food and Agriculture Organization of the United Nations (FAO), 00153 Rome, Italy; melba.reantaso@fao.org; 4 Centre for Sustainable Aquaculture Futures, Biosciences, Faculty of Health and Life Sciences, University of Exeter, Stocker Road, Exeter EX4 4QD, UK; c.r.tyler@exeter.ac.uk

**Keywords:** farm management, antimicrobial resistance, biosecurity, sustainable aquaculture

## Abstract

**Simple Summary:**

In major carp farming regions in Bangladesh, around half of the 231 farms assessed reported disease outbreaks, with the factors influencing disease including geographical region, species stocked, and biosecurity practices. Surveys indicated appropriate disinfection measures were highly successful in preventing diseases, but more generally, there was widespread improper use of chemicals and antibiotics. The study underscores the importance of enhanced in-country training and awareness programs to address biosecurity challenges, in turn ensuring the well-being of farmers and communities while promoting sustainable aquaculture.

**Abstract:**

Small-scale carp polyculture plays a key role in food supply in Bangladesh. However, factors including water pollution, limited infrastructure, and inadequate disease management hinder its sustainability. This paper reports on a survey of 231 farmers across the six major carp producing regions in Bangladesh, analyzing factors including farmers’ social aspects, farm characteristics, information on disease and approaches adopted to combat them, and biosecurity practices. Almost half (46.8%) of the farms surveyed experienced disease in carp species, with clear regional variations. Eighty-four percent of farms reported carp mortalities during disease outbreaks, with an average mortality level of 10.23 ± 11.81%. Clinical signs during outbreaks lasted between a week and a month, with a peak in disease outbreaks occurring in two seasonal periods between June and July and October and December. Disease incidence was related to a range of factors including the farmer’s experience, ponds/farm type, stocked species, and biosecurity practice. A combination of disinfecting measures during pond preparation and measures during stocking, including discarding fingerling transport water away from the farm, fingerling disinfection, and checking the health of fingerlings before stocking, significantly reduced disease occurrence. Treatments involving antibiotics, ciprofloxacin, erythromycin, and azithromycin were reported as ineffective, raising concerns about their non-prudent use, inadequate dosing (perhaps without appropriate veterinary guidance), and the potential for driving antimicrobial resistance in the environment. The research unveils a concerning pattern of high disease incidence across small-scale carp farms in Bangladesh, and the significant potential for disease spread highlights the need for responsible disposal practices. The study emphasizes the need for improving training and awareness programs for addressing biosecurity and disease management challenges, ensuring sustainable aquaculture and community well-being.

## 1. Introduction

Small-scale aquaculture plays a crucial role globally in contributing to food security, poverty alleviation, and livelihoods in numerous nations and in meeting several criteria of UN sustainable development goals (SDGs) [1]. Asia leads in small-scale aquaculture production, with the countries of China, Bangladesh, India, Vietnam, and Indonesia being the major contributors to this production [2]. Small-scale aquaculture is also expanding rapidly in Africa due to the need to alleviate poverty and satisfy the rising demand for fish [3]. Egypt, Nigeria, Uganda, and Kenya are among the nations that are investing heavily in small-scale aquaculture, focusing on tilapia and catfish in particular [3]. In Latin American countries, including Bolivia, Colombia, and Paraguay, small-scale aquaculture accounts for more than 60% of national aquaculture production [4]. Sustainable practices are being promoted to help ensure small-scale aquaculture’s long-term viability. However, small-scale aquaculture faces challenges globally, including limited access to capital, insufficient infrastructure, a lack of technical knowledge, and market constraints.

Endowed with abundant water resources, Bangladesh has become a major contributor to global fish production. The production yield in Bangladesh in fiscal year 2021–22 was 4.62 million metric tons, placing it as one of the world’s foremost fish producers [5]. Aquaculture contributes 57.1% of the nation’s total fish production [5]. The State of World Fisheries and Aquaculture report published in 2022 by the Food and Agriculture Organization (FAO) ranked Bangladesh as the fifth largest aquaculture producer in the world [6]. The fisheries resources of Bangladesh are divided into three distinct categories: inland culture, inland capture, and marine capture. Among these, inland culture fisheries are the most common, including pond/ditch farming, baor (oxbow lake) fishing, shrimp/prawn farming, seasonal cultured water bodies, and pen and cage cultures. Freshwater and coastal aquaculture are the two most common forms of aquaculture in Bangladesh, and both frequently use polyculture systems. Currently, the top seven finfish species cultured in Bangladesh are pangas (*Pangasius pangasius*), tilapia (*Oreochromis niloticus*), rui (*Labeo rohita*), silver carp (*Hypophthalmichthys molitrix*), mrigal (*Cirrhinus cirrhosis*), catla (*Catla catla*), and climbing perch (*Anabas testudineus*), with respective quantities of 447,054 MT, 320,963 MT, 250,046 MT, 193,967 MT, 178,391 MT, 165,244 MT, and 49,659 MT [5]. This information highlights the importance of these species to Bangladesh’s aquaculture industry and their contribution to the country’s overall fish production.

Bangladesh relies heavily on small-scale aquaculture to sustain the livelihoods and food security of a significant portion of its population [7,8,9]. The sustainable development of these aquaculture systems also has enormous potential for addressing environmental concerns associated with the sector and enhancing socioeconomic benefits related to pond production, food security, rural livelihoods, and female empowerment [10,11]. However, the aquaculture industry in Bangladesh faces several sustainability challenges, including those associated with water pollution, limited access to high-quality stock (improved fish strains, etc.) and feed, knowledge gaps regarding biosecurity practices and diseases, and inadequate access to disease diagnostics and health management. There is, furthermore, a lack of real-time information and a comprehensive database regarding the challenges and issues encountered by small-scale carp farmers in Bangladesh. To address this knowledge gap, the aims of the present study were as follows: 1. to assess disease prevalence in small-scale carp farms in Bangladesh and identify the contributing factors to disease occurrence; 2. to evaluate the adoption and effectiveness of biosecurity practices within the small-scale carp farming community; and 3. formulate recommendations for fostering sustainable aquaculture practices, addressing disease-related issues and improving health management, for supporting Bangladesh’s small-scale carp farming industry.

## 2. Materials and Methods

### 2.1. Questionnaire and Online Survey Tools Development

A questionnaire was developed to gather the required information from farmers addressing various aspects of their farming practices, and this included information about the farmers themselves, the farms, stocking data, the farmers’ general observations on diseases, mortality information, and biosecurity practices (Supplementary Materials). The farmers’ observations of clinical symptoms and behavioral changes in fish, whether accompanied by mortality or not, were considered as reports of disease conditions. We also gathered data on unusual fish mortality from farmers who reported a sudden or gradual increase in mortality, accompanied by signs typical of clinical disease. The highest, lowest, and average daily mortality, as well as the number of days of ongoing mortality, were used to compute overall farm mortality levels (%). The absolute total mortalities for a given farm was then calculated by multiplying all categories by the number of days with continued mortalities and used to derive the relative percentage losses from initial stock. Multiple pilot tests were conducted with farmers to ensure the effectiveness and clarity of the questionnaire, and necessary refinements were made based on their feedback. The survey questionnaire was implemented using the “Kobo Toolbox” platform (https://kf.kobotoolbox.org, accessed on 31 December 2021), which was chosen as an online tool designed specifically for data collection and gathering. Kobo Toolbox provides a range of tools and features for creating and administering surveys or questionnaires, collecting data from diverse sources, and managing data efficiently. One significant advantage of the Kobo Toolbox platform is its capability for offline data collection, which is particularly valuable in areas with limited or unreliable internet connectivity. Mobile devices such as smartphones or tablets can be employed to download the survey forms, enabling offline data collection. Once an internet connection became available, the collected data could be uploaded to the Kobo Toolbox server. The platform also facilitated the secure storage and management of the collected data, allowing for data exports in various formats such as Excel or CSV to facilitate the subsequent analysis.

### 2.2. Case Definition of Small-Scale Carp Farmer

Small-scale carp farmers are defined as those who engage in a low-input and low-output fish farming practice with a maximum farm size of 2 hectares. They rely on aquaculture in primary or secondary manners to support their livelihood and generate household income and cultivate carp species, either exclusively or in combination with other species, with a minimum carp stocking proportion of 20% of the total stock.

### 2.3. Study Area and Farm Selection

**Figure 1:**
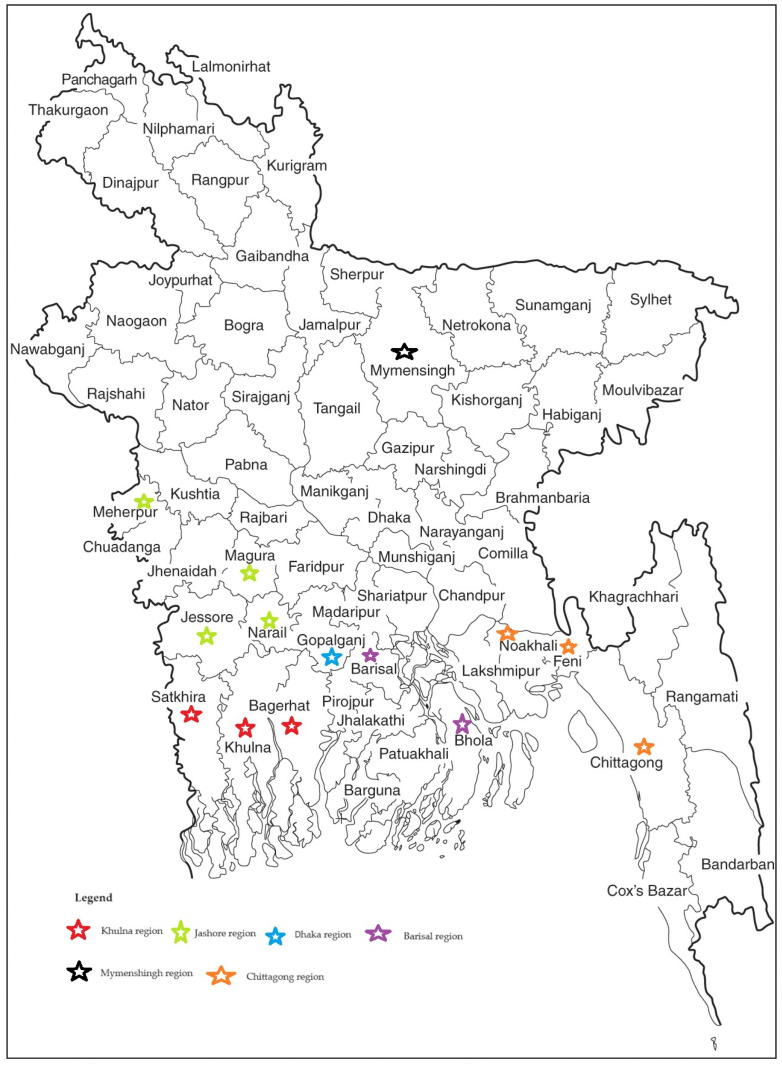
The location of the farms in each of Bangladesh’s six study regions: Barisal, Chittagong, Dhaka, Khulna, Mymensingh, and Rajshahi.

Based on aquaculture production data obtained from the Department of Fisheries [12], the top six carp-producing regions in Bangladesh, namely Barisal, Chittagong, Dhaka, Jashore, Khulna, and Mymenshingh, were selected for this research (Figure 1). A primary census list containing 998 known farms with at least 20% carp as the crop species was compiled. This list included information such as the farmer’s name, mobile phone number, and farm identification to ensure accurate farm identification. The total number of farms from the census list was used to determine the sample size for the final survey, aiming for a 95% confidence level, using a sample calculator software (https://www.calculator.net/sample-size-calculator.html, accessed on 2 January 2022). Subsequently, 278 farmers were randomly selected from each of the six regions to create a representative sample using an online randomizing tool (https://www.randomizer.org, accessed on 2 January 2022). Recognizing that some farmers may be unreachable or hesitant to participate, previous surveys estimated that up to 30% of farmers might fall into this category. With the aim of resolving this issue, a random list of alternative farms was compiled from the database and used as applicable/required.

### 2.4. Survey Implementation

Four skilled enumerators were engaged to carry out this survey, receiving comprehensive training from the project leader on the questionnaire and survey procedures. Subsequently, these enumerators conducted the surveys between January and April 2022. Prior to gathering information, the consent of the farmers was obtained, ensuring their willingness to participate and that ethical practices were upheld. The data regarding farms and farmers were collected with their explicit permission, while farmers provided information related to diseases, clinical symptoms, and mortality based on their recollection of the events and established knowledge. A comprehensive survey was conducted across six regions, involving interviews with a total of 281 farmers. Following the surveys, the gathered data were stored on tablets to enable offline scrutiny by enumerators and project leads. After a thorough review of the data, the completed survey documents were subsequently uploaded to the online database of the Kobo-Toolbox platform for further analysis and accessibility.

### 2.5. Data Gathering: Preparation and Analysis

We retrieved all survey data from the Kobo platform in Excel format and then meticulously reviewed the information. Out of 281 farms for which data were collected, information from 50 farms was excluded from the final analysis because these farms did not meet the criteria for small-scale carp farms, such as having a farm size larger than the defined case definition. Before conducting the analysis, the data were subjected to a series of procedures to ensure their accuracy and enhance their utility. The variables were analyzed and, where applicable, merged to produce biologically relevant groups, avoiding small categorical groups with fewer than ten entries. For instance, the primary and secondary occupations were merged into broader categories. Business-related occupations such as business, contractor, feed merchant, and shopkeeper were classified as “business”, whereas smaller groups such as students, housewives, and immigrants were classified as “others.” Similarly, the water data source was organized according to various combinations: ground, river/canal, ground + river/canal, and rain + nearby farm. Stocking species also underwent grouping into the following categories. ‘Carp’ included Rohu, Catla, Silver carp, Mrigal and Grass carp (*Ctenopharyngodon idella*). ‘Tilapia’ was retained as a separate group given its significance in carp polyculture. Freshwater species other than carp and tilapia were grouped as “other freshwater species which includes Pangas, walking catfish (*Clarias batrachus*), Stinging catfish (*Heteropneustes fossilis*), Golsha tengra (*Mystus bleekeri*)”, while brackish water species, including seabass (*Lates calcarifer*), shrimp (*Penaeus monodon*), mullet (*Mugil cephalus*), and others, were grouped as “brackish water species”.

Data processing was carried out using Excel MS Office 2019, and the subsequent analysis was performed using SPSS (IBM SPSS Statistics 22). The Pearson chi-squared test and Fisher’s exact test assessed statistical differences between farms that reported diseases or unusual mortality (data on unusual mortality was collected for farmers reporting any rapid or steady increase in fish mortality with the typical clinical signs of diseases) and those that did not (a yes/no, binomial variable). The Kruskal–Wallis and Mann–Whitney U tests analyzed factors significantly associated with reported mortality, measured as a continuous variable (percentage). As a criterion to determine whether the observed differences were statistically significant, a *p*-value of 0.05 was used.

The analysis involved an initial data assessment, considering the number of observations and biological plausibility to identify potential confounders for disease occurrence and unusual mortality. Various characteristics related to farmers’ social aspects, farm and farming practices, and biosecurity practices were analyzed for their impact on disease occurrence. Logistic regression or generalized linear models were used for the analysis, evaluating the effect of confounding variables on observed differences in disease occurrence. Confounding factors with a *p*-value of 0.2 in the univariable regression analyses were included in the multivariable regression, employing backward selection followed by forward selection with a significance level of 0.05. Odds ratios (ORs) were calculated to measure the strength and direction of associations between variables in the regression models, with 95% confidence intervals (CIs) computed to assess the precision of estimates and determine statistical significance. The Akaike information criterion (AIC) was utilized to select the final model. The statistical analyses were performed using R, version 4.3.0.

## 3. Results

### 3.1. Farmer Profile: Social and Regional Perspectives

This study conducted a comprehensive survey of 231 small-scale carp farms (n = 231) across six regions of Bangladesh, namely Barishal, Chittagong, Dhaka, Khulna, Mymenshingh, and Jashore (Figure 1). The Barishal region had the maximum proportion of interviewed farms, 28.6% (n = 66), while the Dhaka region had the lowest representation, 7.8% (n = 18). All interviews were conducted solely with farm owners, with 2.2% (n = 5) of the participants being female and the remainder male. The small-scale carp farmers varied in their age, educational background, and farming experience (Table 1). The average age of the farmers was 45 years, with Chittagong having the highest average age (50.8 years) and Khulna the lowest (41.5 years). Educational backgrounds exhibited variation, with 15.2% having no formal education, 50.6% up to the higher secondary level, and 15.2% possessing a university degree. Among the surveyed farmers, 62.3% identified aquaculture as their primary occupation, with no significant regional variations in both primary and secondary occupations. The average farming experience was 15 years, with a significant variation in farming experience within the regions (Table 1).

### 3.2. Farm Characteristics and Practices

The analysis of farm characteristics illustrated significant regional variations in farm size, pond numbers, water source preferences, stocking density, and the percentage of average carp stocked in carp polyculture farming (Table 1). The average farm size was 0.69 ± 0.46 hectares, with Jashore having the largest farms (0.83 hectares ± 0.46) and Dhaka the smallest (0.58 ± 0.24 hectares). Groundwater together with rain was the primary water source preference (44.2%, n = 102). Polyculture was practiced across all farms, with diverse species combinations and no significant regional differences. Jashore (44.8%, n = 26) and Chittagong (50%, n = 12) predominantly practiced carp polyculture with tilapia, while Dhaka (66.7%, n = 12) and Barisal (54.5%, n = 36) focused on carp polyculture with tilapia and other freshwater species. Coastal regions (Barisal and Khulna) incorporated brackish water species in carp polyculture. Fingerling sourcing varied, with 37.7% (n = 87) obtaining from hatcheries. Stocking density per hectare showed regional variations, with Mymensingh having the highest (366,756 fingerlings/ha) and Chittagong the lowest (39,495 fingerlings/ha). The average stocking percentage with carp species was 45.6%, with Chittagong having the highest (60.1%) and Dhaka the lowest (37.9%) (Table 1).

### 3.3. Disease Incidence and Pattern

Forty-seven percent (n = 108) of farms indicated diseases occurrence in carp species throughout the farming year. The prevalence of diseases showed significant variation among different farming regions (*p* < 0.001), with the Chittagong region reporting the highest incidence (95.8%, n = 23) and the Khulna region the lowest incidence (22.7% n = 10) (Table 2). Among the farms with disease outbreaks, 84.25% (n = 91) reported associated mortality in the carp, with an average mortality level of 10.23 ± 11.81%. Conversely, 15.75% of farms reported disease incidence without any associated mortality. The highest mortality level was observed in Barisal (10.3%), while Mymensingh had the lowest (6.5%). However, there were no significant differences (*p* = 0.418) in mortality levels across the farming regions.

**Figure 2:**
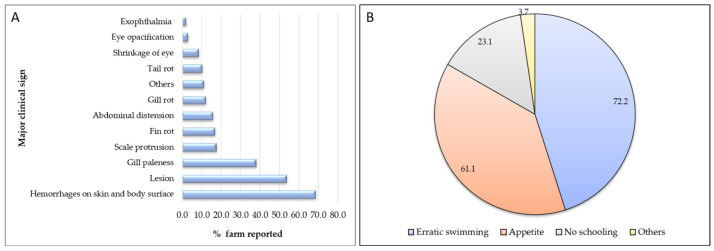
Aspects relating to reported disease incidence on small carp farms in Bangladesh: (**A**) incidence of major clinical signs of diseases reported by farmers, expressed as a percentage; (**B**) major behavioral changes reported by farmers, expressed as a percentage.

Farmers documented various clinical signs and observed behavioral changes during disease outbreaks. The most commonly reported clinical signs were hemorrhages on the skin and body surface (68.5%), followed by lesions (53.7%), gill paleness (38%), scale protrusion (17.6%), fin rot (16.7%), and abdominal distension (15.7%) (Figure 2). Similarly, the most prevalent behavioral change reported by 72.2% of farmers was erratic swimming, while a decrease in appetite was reported by 61.1% of farmers (Figure 2).

**Figure 3:**
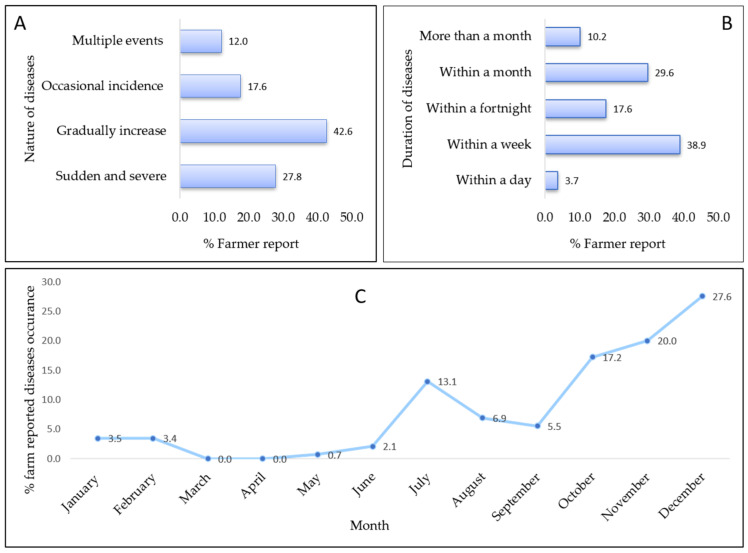
Diseases patterns in small-scale carp farming in Bangladesh: (**A**) type of diseases/mortality incidence/progression; (**B**) duration of diseases or mortality events; (**C**) seasonal patterns of disease occurrences.

Considering the duration of diseases or mortality events, 38.9% of farmers reported occurrences extending for periods for over a week, 17.6% for two weeks, and 29.6% for over a month (Figure 3). Mortality levels were 12.3%, 12.2%, and 10.2% where disease durations were extended for a month or more, within a week, and within a fortnight, respectively. Regarding the nature of disease incidence or mortality, 42.6% of farmers reported a gradual intensification, while 12% reported multiple events of the same diseases (Figure 3). Notably, the mortality level was highest (22.3%) for multiple events of the same apparent disease. There was a significant difference (*p* < 0.001) between the nature of the disease or mortality and the level of mortality. In terms of the seasonality of disease incidence, our analysis revealed clear patterns in disease occurrence throughout the year, with two prominent peaks: one extending from June to July and another from October to December (Figure 3) and the highest incidence occurring in December, accounting for 27.6% of reported cases (n = 40). Conversely, there were no reported diseases in both March and April. No farm reported sending diseased samples for laboratory diagnosis for disease outbreaks or mortality events.

### 3.4. Diseases Management and Treatment

**Figure 4:**
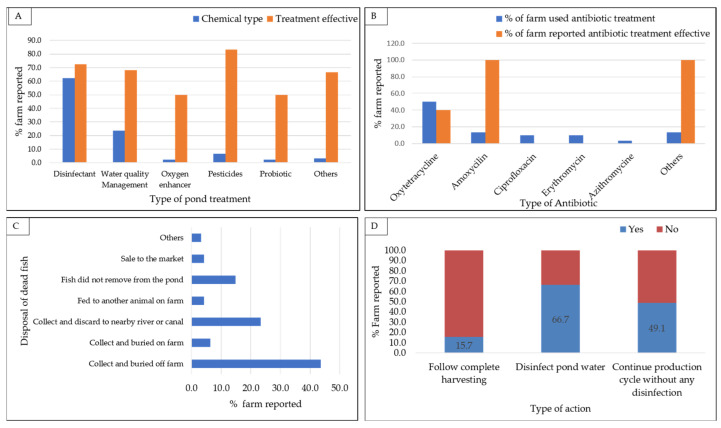
Treatments implemented and management actions in response to disease outbreaks: (**A**) use and efficacy of different pond treatments in combating diseases; (**B**) effectiveness of different antibiotics for disease control; (**C**) types of action for dead fish disposal: (**D**) farm’s post-outbreak disease management strategies.

During disease outbreaks, farmers employed various chemical and antibiotic treatments. A total of 86% (n = 93) of farms applied chemical treatments during outbreaks, with 71% (n = 66) reporting effectiveness in controlling disease incidence. Notably, disinfectants were the most frequently used chemical treatments (62.4% of farms, n = 58) (Figure 4). Regarding antibiotic treatment, among the 108 disease-reporting farms, 27.8% (n = 30) used antibiotics for treatment, but only 46.7% (n = 14) of these farms reported antibiotic treatment as effective in gaining any fish recovery. Oxytetracycline was the most commonly used antibiotic, used by 50.0% (n = 15) of farms applying antibiotic treatment, with a documented effectiveness of only 40.0% (n = 6). However, ciprofloxacin, erythromycin, and azithromycin used by 10.0%, 10.0%, and 3.3% of farms, respectively, were reported to be ineffective in treating disease states (Figure 4).

Regarding disease outbreaks, the study explored the adoption of management practices among farmers, and notably, the proper disposal of deceased fish emerged as a crucial aspect of maintaining farm biosecurity. Among farms affected by diseases leading to fish mortality, the majority (43.6%, n = 41) practiced collecting and burying dead fish off the farm, while 23.4% (n = 22) opted for disposing of them in nearby rivers or canals. Remarkably, 14.9% (n = 14) of the farms reported not discarding dead fish from their ponds (Figure 4). Likewise, only 15.7% (n = 17) of farms reported implementing the practice of complete harvesting, with the majority (84.3%) neglecting this measure, posing a potential risk of disease persistence. Although disinfecting pond water was a widely adopted biosecurity measure, approximately 33.3% of farms omitted this step, which would increase the chance of pathogen persistence at a farm and the spread of disease. Interestingly, about half of the disease-affected farms (51.0%, n = 55) proceeded to their next production cycle without implementing any disinfection measures, indicating a potential gap in good biosecurity practice (Figure 4).

### 3.5. Factors Associated with Disease Incidence

Results pertaining to farmers’ social aspects revealed significant associations between farmers’ occupations (*p* < 0.001) and farming experience (*p* = 0.003) with disease occurrence (Table 2). Aquaculture-focused farmers exhibited a lower disease incidence of 36.8% (n = 53), compared with those in other occupations (Table 2). However, a notable findings surfaced regarding farming experience, with the highest percentage (58.7%) of farmers reporting disease incidence having farming experience exceeding 20 years, while the lowest percentage (23.1%) of farmers reporting disease incidence had farming experience ranging from 1 to 5 years. Moreover, there were significant differences in disease incidence reporting among the different farming experience groups. (Table 2). However, no significant associations were found between farmers’ age (*p* = 0.069) and education (*p* = 0.760) and disease incidence (Table 2).

Various factors related to farms and farming practices showed significant associations with disease incidence (Table 2). Farms with more ponds per farm (*p* =0.006) reported a higher disease incidence, but no significant association was found with farm size (*p* = 0.211) and disease incidence. There was no significant association between the water source used by the farms with disease incidence (*p* = 0.060; Table 2). Concerning fish stocking, there was a significant association identified between the combination of stocked species (*p* < 0.001), source of fingerlings (*p* = 0.038), and the proportion of carp in the stocked pond (*p* = 0.049) with disease (Table 2). Higher stocking densities also seemed to provoke diseases, though the association was marginally non-significant (*p* = 0.057). Farms stocking a combination of carp, tilapia, freshwater, and brackish water species reported a higher disease incidence (78.6%, n = 22) than those with a combination of carp and tilapia (56.1%, n = 37). Farms stocking only carp species had the lowest disease incidence at 26.7% (n = 4). Regarding fingerling sources, farms using hatchery-supplied fish (68.8%, n = 22) (which also brings in a further water source) reported the highest disease incidence, while farms using nursery-sourced fingerlings reported the lowest incidence (37%, n = 10) (Table 2).

An analysis of biosecurity practices during pond preparation revealed farmers employed various pond cleaning measures, including having a fallow period, drying the pond bed, bleaching, and liming. The length of the fallow period, spanning from without any interval to over 30 days, demonstrated a tendency for reductions in disease occurrence with the fallow period, but this was not statistically significant (*p* = 0.069). However, combinations of the disinfecting measures during pond preparations were associated with reduced disease incidence (*p* < 0.001; Table 3). On farms adopting a combination of pond bottom drying, bleaching, and liming, there was no reported disease incidence (Table 3). The introduction of new stock was significantly associated (*p* = 0.005) with disease incidence (Table 3). Similarly, combining biosecurity measures related to fish stocking was significantly associated (*p* = 0.010) with disease incidence (Table 3). Farms implemented different combinations or only one of the biosecurity measures such as disposing of transport water off-farm, disinfecting fish, and conducting health checks during fish stocking. The most successful way to avoid diseases related to bringing fish onto a farm (77.8%, n = 7) was where the fish transport water was disposed of off the farm and fish were disinfected before stocking (Table 3). Among general biosecurity practices during farming, the restriction of the entrance of domestic or other animals into the farm was significantly associated (*p* = 0.002) with disease incidence, with farms following this practice experiencing the lowest incidence (27.8%, N = 15) (Table 3). Conversely, a farm perimeter fence (*p* = 0.408), restricting the entrance of people generally into the farm (*p* = 0.440), shared equipment with other farms (*p* = 0.889), hired harvesters (*p* = 0.479), and hired harvesting equipment (*p* = 0.565) showed no significant association with disease incidence (Table 3).

A multivariable model regression analyses found that the region (Jashore, Khulna, and Mymenshingh), farming experience, number of ponds on the farm, carp as a proportion of the total fish stocked in a pond, the combination of species stocked (carp along with tilapia and other freshwater species, carp with tilapia, the combination of carp, tilapia, fresh- and brackishwater spp.), and biosecurity measures during fish stocking were all significantly associated with the occurrence of diseases being reported (Table 4 and Table 5).

### 3.6. Farmers’ Observations Regarding Diseases

**Figure 5:**
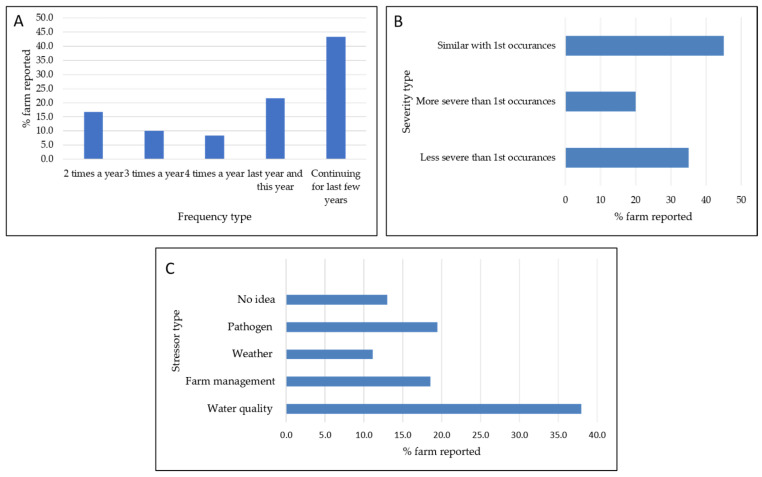
Farmers’ observations regarding diseases outbreaks: (**A**) frequency of similar disease repetitions, (**B**) severity of the repeated diseases compared with the first occurrence, (**C**) farmers’ observation about stress factors believed to be associated with disease occurrences.

The questionnaire also captured factors perceived by farmers relating to disease patterns, stressors, disease recurrence, and the intensity of recurrent diseases. Among farms affected by diseases, 35% (n = 38) reported that the initial disease outbreak was believed to have originated from nearby farms (data not included). Within this subset of farms affected by disease, 71% (n = 27) of farmers observed similar disease conditions as those occurring on their neighboring farms, indicating likely disease transmission. Regarding stressor factors in farm management, features of poor water quality were the most prevalent, reported by 38% (n = 41) of farmers (Figure 5). Notably, 13.0% (n = 14) of farmers reported having “No Idea” about what constituted possible stressors to the fish on their farms, suggesting a need for greater awareness and understanding of potential stressors in farm management (Figure 5). Among farmers who encountered diseases on their farms, 55.6% (n = 60) reported repeated occurrences of diseases that appeared to be similar in nature. The highest frequency of recurrence of apparently similar disease conditions was reported at a level of 43.3% (n = 26) for that happening over a period of a few years, whilst four repetitions of apparently the same disease condition within a single year were reported by 8.3% of farmers (n = 5) (Figure 5). Regarding the severity of diseases in cases of repeated occurrence, indicated in 20.0% of these cases (n = 12), farmers reported that the diseases were more extreme in the second versus the first outbreak. (Figure 5).

## 4. Discussion

This study assessed disease prevalence in relations to farm operation and biosecurity practices for the purpose of proposing strategies for enhancing productivity for small-scale carp polyculture farming in Bangladesh. The findings of this study highlight the significant challenge faced by farmers in maintaining the health of the carp species cultured, with approximately half of the farms (46.8%) reporting the occurrence of disease during the farming year. Disease occurrence varied across different farming regions, with the highest incidence occurring in Chittagong and the lowest in Khulna, indicating local factors related to environmental conditions and management practices may contribute to disease prevalence. Among farms experiencing disease outbreaks, although there were differences in cases of disease incidences across regions, the majority of mortality levels were relatively consistent. These rates of disease are reasonably consistent with some previous studies reporting for carp species in Bangladesh (e.g., [13], 13.8%,) and somewhat lower than for others (29.6%; [14]). These levels of losses, however, highlight the urgency for proactive measures and interventions to address the growing challenges posed by fish diseases for carp (and other finfish species) aquaculture in Bangladesh. The majority of farmers (55.6%) experienced repeated occurrences of apparently similar disease condition outbreaks on their farms, indicating common underlying contributing factors. Clinical (and behavioral) signs described by the farmers, the nature and duration of diseases, and the seasonality of outbreaks align with previous research on carp diseases and indicate the presence of several viral, bacterial, or fungal diseases in small-scale carp farming in Bangladesh. However, none of the infected farms had samples sent to any laboratories for disease diagnosis, due in part to a lack of knowledge about where and how to send them.

In the farm survey, it was found where disease conditions were more prolonged on the farm (those exceeding a month), and these were associated with the highest mortality rates, and not surprisingly, multiple events of what appeared to be a similar disease condition had the highest mortality level. The seasonality in the reported disease outbreaks (in June to July and October to December) are also in-keeping with previous findings in Bangladesh corresponding to the rainy and winter seasons [13,14]. Disease outbreaks occurring in winter likely relate to reduced pond water levels and in summer due to heavy rains, both of which can lead to degraded water quality [15,16]. Temperature has also been shown to have a critical role in some carp disease outbreaks in Bangladesh [17].

A common response to disease outbreaks by farmers in this study was the use of chemicals, as has been reported previously in Bangladesh [13,14,17]. In our survey, disinfectants were most commonly used to combat disease outbreaks (62.4% of farmers), and this showed a high success rate, with 72% reporting this treatment as effective in recovering their farms from disease. Interestingly, 30% of farms reported that chemical treatment was ineffective in controlling diseases, which may bring about the question of misunderstandings or misuses of chemicals in disease management, and this supports a previous paper reporting a limited understanding of dosage effectiveness and potential side effects of chemical treatment systems applied in farm settings in Bangladesh [17,18,19].

Regarding antibiotic usage, the study revealed a high application level for disease treatment (27.8% of the farms with disease), contrasting sharply with earlier reports of levels of between only 5.5 and 6.15% (26, 14, respectively). Furthermore, only 46.7% of farms reported any effectiveness of antibiotic treatment, which is somewhat lower than in a previous study, at 72% [15]. For oxytetracycline, the predominant antibiotic used (50.0% of farms), only a small fraction of farms experienced positive results, and for ciprofloxacin, erythromycin, or azithromycin treatments, these were reported to be ineffective. Thus, we identify that either there was wrong/inappropriate antibiotic selection for treating specific diseases and/or poor applications, raising further concerns about antibiotic misuse and the emergence of antimicrobial resistance, which in turn poses a potential threat to the long-term sustainability of aquaculture. The limited availability of veterinarians or aquatic animal health professionals to provide the right advice further compounds this problem [17].

In addition to direct chemical treatments, a range of diverse management practices are operated by the farms to recover and minimize the further spread of the diseases. The study found that around half the farms (47.6%) practiced collecting and burying dead fish off the farm as a means of proper disposal. However, almost one quarter of the farms (23.4%) disposed of their dead fish in nearby rivers or canals, potentially posing a risk of disease transmission to the surrounding aquatic environment. Furthermore, the fact that most farms did not undertake complete harvesting after a disease outbreak means a more enhanced likelihood of major diseases in the subsequent crop. A thorough cleaning of ponds after a disease outbreak is considered essential to avoid/minimize subsequent disease likelihood. The failure to operate this in so many carp farms further emphasizes the need for increased awareness and education among farmers to ensure this is implemented for good biosecurity measures for mitigating disease risks and maintaining the health of aquaculture operations.

Among farmers’ social aspects, occupation and farming experience was found to be significantly associated with disease incidence. Farmers primarily focused on aquaculture had lower disease rates on their farms compared to those with alternative occupations, and those farmers solely relying on aquaculture for their income had the lowest disease incidence also. Interestingly, however, the relationship between farming experience and disease occurrence yielded results that are hard to explain, with a longer experience associated with a higher disease incidence. This may, however, relate to farmers coming more recently into carp production having better expertise in disease management and farm-level biosecurity. Contrary to several earlier studies suggesting a potential association between water sources and disease incidence, the current study found no significant association between water sources and disease incidence in small-scale carp farms [15].

Among fish stocking practices, species combination, the source of fingerlings and the stocking percentage of carp species were found to be significantly associated with disease incidence. Farms stocking a combination of carp, tilapia, freshwater, and brackishwater species (Khulna region) had a significantly higher disease incidence than farms stocking other combinations. Carp are typically freshwater species, and when placed in saline water conditions (as occurs with shrimp and prawn farms), this imposes a heightened level of physiological stress, potentially increasing their susceptibility to diseases. However, over centuries, China has developed carp polyculture as a sustainable aq-uaculture practice [20,21]. In this study, where farmers exclusively stocked carp species, there was a significantly lower disease incidence compared to other stocking combinations. This could be attributed to findings from prior research indicating that intensive culture and mixed stocking may exacerbate disease outbreaks [22,23]. Most farmers source fingerlings from a combination of hatcheries and nurseries or exclusively from either hatcheries or nurseries, aligning with findings from previous studies [15,16,19,24]. Of concern in the data recovered was that 13.9% of farms mention acquiring fingerlings with incoming water from sources with a higher incidence of diseases, raising further biosecurity concerns.

Adopting proper sanitary conditions plays a vital role in good pond farming practice, and this is cost-effective and relatively easy to implement [25]. Indeed, undertaking pond preparation, practices such as draining, cleaning, liming, and drying are widely recommended before the next production cycle [26]. In the analyses of the study farms, measures during pond preparation were significantly linked to disease incidence. Farmers employing a combination of pond bed drying, bleaching, and liming reported no disease incidence, whereas those adopting less stringent measures suffered diseases. Thus, from this extensive analysis of farms across many regions in Bangladesh, we show that good measures for pond preparation can be highly effective in preventing diseases in small-scale carp farming. Similarly, adopting the measures of discarding fish transport water off the farm, refraining from combining new stock with existing stock, and restricting entrance for domestic animals, can all serve to lower disease risk/incidence.

The observations and perceptions of farmers in this study offer valuable insights into the disease dynamics and biosecurity practices of small-scale carp farming in Bangladesh. The study reveals that over a third (35%) of farms affected by diseases reported that the initial outbreak originated in nearby farms, which is reinforced by 71% of farmers indicating similar disease states on their farms to neighboring farms. This finding supports the likelihood for localized farm transmissions of diseases and, for one thing, the need to avoid the current common practice of disposing dead fish into nearby open water bodies. Relating to water also, water quality clearly (and how this is affected by seasons and climatic patterns) underpins a major aspect of disease incidence in Bangladesh. This emphasizes the importance of ensuring a supply of clean and suitable water sources for farming, and this is arguably one of Bangladesh’s greatest challenges for aquaculture, which will be further exacerbated with climate change and increasing demands for freshwater from diverse industry, urban, and agriculture sectors.

Through this extensive survey of six of the major finfish-producing regions in Bangladesh, we discovered that the inappropriate use of chemicals and antibiotics could potentially contribute to the severity of diseases, raising concerns also regarding antimicrobial resistance. Consequently, there is an urgent need for enhanced training and awareness programs to assist farmers in identifying and addressing the key challenges associated with ensuring proper biosecurity and disease management. Similarly, a previous study proposes that improvements in existing aquaculture practices can be achieved through the assistance of animal health professionals, financial aid, training in proper farm management, and the provision of necessary chemicals/medicines by relevant authorities [27]. This, in turn, will contribute to sustainable aquaculture and the overall well-being of farmers and their communities.

This research activity had several limitations worth noting. Due to the COVID-19 pandemic, face-to-face data collection had to be avoided, potentially impacting the depth of information obtained, and no economic analysis was conducted due to the combination of time constraints and the challenge of collecting financial data over the phone. Furthermore, the absence of biological sample collections and laboratory analyses limited the validation and accuracy of reported disease cases. A diagnosis of the specific disease reported would help in providing a more comprehensive understanding of how and why these conditions arise and in turn better inform appropriate biosecurity practices for small-scale carp farming.

## 5. Conclusions

This study on small-scale carp polyculture farming in Bangladesh revealed significant challenges in disease management, with approximately half of the farms experiencing disease outbreaks. Regional variations in disease occurrence underscored the influence of local environmental conditions and management practices. Recurring disease outbreaks were common and often prolonged and were related to inadequate biosecurity measures, with farmers resorting to chemical and antibiotic treatments, albeit with varying efficacy. Social factors such as occupation and farming experience were associated with disease incidence, while stocking practices and pond preparation significantly influenced disease prevalence. Collaboration with animal health professionals and relevant authorities is imperative to address both the issues of accurate disease diagnosis and the emerging concerns of antimicrobial resistance, vital for enhancing sustainable aquaculture practices in Bangladesh.

## Figures and Tables

**Table 1:** Regional characteristics of farmers and farms.

Characteristics	Region (Number of Farms Studied)						
	Barishal (66)	Chittagong (24)	Dhaka (18)	Khulna (44)	Mymenshingh (21)	Jashore (58)	
	n (%) or Mean (Min, Max)						*p*
**Farmer Age (Years)**	46.0 (25–75)	50.8 (30–65)	42.2 (24–65)	41.5 (23–65)	42.2 (29–65)	46.2 (30–70)	0.008
**Farmer Education Level**							
No Education	10 (15.2)	3 (12.5)	6 (33.3)	5(11.4)	4 (19.0)	7 (12.1)	0.008
Primary (1–5)	21 (31.8)	9 (37.5)	1 (5.6)	4 (9.1)	3 (14.3)	6 (10.3)	
Higher Secondary (6–12)	28 (42.4)	11 (45.8)	8 (44.4)	26 (59.1)	11 (52.4)	33 (56.9)	
University (Bachelor/MS/More)	7 (10.6)	1 (4.2)	3 (16.7)	9 (20.5)	3 (14.3)	12 (20.7)	
**Primary Occupation**							
Agriculture	10 (15.2)	9 (37.5)	1 (5.6)	5 (11.4)	0 (0.0)	1 (1.7)	0.729
Aquaculture	37 (56.1)	3 (12.5)	16 (88.9)	33 (75.0)	16 (76.2)	39 (67.2)	
Business	6 (9.1)	7 (29.2)	1 (5.6)	2 (4.5)	4 (19.0)	10 (17.2)	
Job/Service	12 (18.2)	4 (16.7)	0 (0.0)	3 (6.8)	1 (4.8)	7 (12.1)	
Others	1 (1.5)	1 (4.2)	0 (0.0)	1 (2.3)	0 (0.0)	1 (1.7)	
**Secondary Occupation**							
No Secondary Occupation	4 (6.1)	0 (0.0)	2 (11.1)	4 (9.1)	3 (14.3)	3 (5.2)	0.428
Agriculture	9 (13.6)	0 (0.0)	4 (22.2)	13 (29.5)	4 (19.0)	20 (34.5)	
Aquaculture	29 (43.9)	21 (87.5)	2 (11.1)	11 (25.0)	5 (23.8)	19 (32.8)	
Business	19 (28.8)	3 (12.5)	4 (22.2)	10 (22.7)	6 (28.6)	10 (17.2)	
Job	2 (3.0)	0 (0.0)	1 (5.6)	0 (0.0)	0 (0.0)	0 (0.0)	
Others	3 (4.5)	0 (0.0)	5 (27.8)	6 (13.6)	3 (14.3)	6 (10.3)	
**Farming Experience (Years)**							
1–5 years	9 (13.6)	0 (0.0)	3 (16.7)	8 (18.2)	3 (14.3)	3 (5.2)	0.004
6–10 years	24 (36.4)	2 (8.3)	6 (33.3)	14 (31.8)	7 (33.3)	15 (25.9)	
11–15 years	15 (22.7)	3 (12.5)	3 (16.7)	10 (22.7)	5 (23.8)	15 (25.9)	
16–20 years	14 (21.2)	5 (20.8)	2 (11.1)	6 (13.6)	3 (14.3)	10 (17.2)	
Above 20 years	4 (6.1)	14(58.3)	4 (22.2)	6 (13.6)	3 (14.3)	15 (25.9)	
**Total Farm Area (Ha)**	0.54 (0.03–1.82)	0.77 (0.13–1.62)	0.58 (0.26–1.01)	0.67 (0.03–2.00)	0.79 (0.08–1.27)	0.83 (0.08–1.68)	0.001
**Total Water Spread Area (Ha)**	0.44 (0.02–1.62)	0.65 (0.01–1.38)	0.46 (0.18–0.81)	0.55 (0.02–1.74)	0.63 (0.07–1.05)	0.65 (0.06–1.47)	0.001
**Number of Ponds in Farm**	3 (1–10)	3 (1–13)	2 (1–5)	2 (1–7)	3 (1–6)	2 (1–4)	<0.001
**Farm type**							
Perennial	63 (95.5)	24 (100)	18 (100)	43 (97.7)	21 (100)	58 (100)	0.096
Seasonal	3 (4.5)	0 (0.0)	0 (0.0)	1 (2.3)	0 (0.0)	0 (0.0)	
**Water Sources**							
Groundwater	7 (10.6)	24 (100.0)	2 (11.1)	18 (40.9)	20 (95.2)	48 (82.8)	<0.001
River/Canal	52 (78.8)	0 (0.0)	12 (66.7)	22 (50.0)	0 (0.0)	3 (5.2)	
Rain	1 (1.5)	0 (0.0)	2 (11.1)	1 (2.3)	0 (0.0)	5 (8.6)	
Groundwater + River/Canal	6 (9.1)	0 (0.0)	0 (0.0)	0 (0.0)	1 (4.8)	2 (3.4)	
Rain + Nearby Farm	0 (0.0)	0 (0.0)	2 (11.1)	3 (6.8)	0 (0.0)	0 (0.0)	
**Stocking Species**							
Carp	0 (0.0)	0 (2.6)	1 (5.6)	2 (4.5)	1 (4.8)	11 (19.0)	0.673
Carp + Tilapia	10 (15.2)	12 (50.0)	5 (27.8)	12 (27.3)	1 (4.8)	26 (44.8)	
Carp + Tilapia + Other freshwater Species	36 (54.5)	12 (50.0)	12 (66.7)	15 (34.1)	8 (38.1)	5 (8.6)	
Carp + Tilapia + Freshwater Species + Brackishwater Species	19 (28.8)	0 (0.0)	0 (0.0)	9 (20.5)	0 (0.0)	0 (0.0)	
Carp + Other Freshwater Species	1 (1.5)	0 (0.0)	0 (0.0)	6 (13.6)	11 (52.4)	16 (27.6)	
**Source of Fingerlings**							
Hatchery	29 (43.9)	9 (37.5)	8 (44.4)	10 (22.7)	6 (28.6)	25 (43.1)	0.504
Nursery	8 (12.1)	0 (0.0)	7 (38.9)	5 (11.4)	4 (19.0)	3 (5.2)	
Hatchery + Nursery	11 (16.7)	15 62.5)	3 (16.7)	16 (36.4)	11 (52.4)	20 (34.5)	
Hatchery + Incoming Water	17 (25.8)	0 (0.0)	0 (0.0)	10 (22.7)	0 (0.0)	5 (8.6)	
Others	1 (1.5)	0 (0.0)	0 (0.0)	3 (6.8)	0 (0.0)	5 (8.6)	
**Stocking Density (Number/Ha)**	97,810 (2059–1,235,500)	39,495 (5343–98,840)	111,239 (15,444–329,467)	105,912 (6919–1,445,168)	366,756 (3802–1,728,635)	60,212 (3756–658,933)	<0.001
**Average % of Carp stocked)**	39.5 (20–85)	60.1 (20–95)	37.9 (24–100)	38.8 (20–100)	38.1 (25–100)	56.6 (20–100)	<0.001

**Table 2:** Association of farmers’ social aspects and farming practices with disease incidence.

	All Farms	Reported Diseases		
	n = 231	Yes (n = 108)	No (n = 123)	
Variables	n (%) or Mean (Min, Max)	n (%) or Mean (95% CI)		*p*
**Region**				
Barishal	66 (28.6)	42 (63.6)	24 (36.4)	<0.001
Chittagong	24 (10.4)	23 (95.8)	1 (4.2)	
Dhaka	18 (7.8)	9 (50.0)	9 (50.0)	
Khulna	44 (19.0)	10 (22.7)	34 (77.3)	
Mymensingh	21 (9.1)	5 (23.8)	16 (76.2)	
Jashore	58 (25.1)	19 (32.8)	39 (67.2)	
**Farmer age**	45 (23–75)	46 (24–65)	44 (23–75)	0.069
**Education**				
No education	35 (15.2)	14 (40.0)	21 (60.0)	0.76
Primary (1–5)	44 (19.0)	22 (50.0)	22 (50.0)	
Higher secondary (6–12)	117 (50.6)	57 (48.7)	60 (51.3)	
University degree	35 (15.2)	15 (42.9)	20 (57.1)	
**Primary occupation**				
Agriculture	26 (11.3)	20 (76.9)	6 (23.1)	<0.001
Aquaculture	144 (62.3)	53 (36.8)	91 (63.2)	
Business	30 (13.0)	16 (53.3)	14 (46.7)	
Job	27 (11.7)	17 (63.0)	10 (37.0)	
Others	4 (1.7)	2 (50.0)	2 (50.0)	
**Farming experience**	15 (1–40)	16 (2–40)	13 (1–40)	0.003
1–5 years	26 (11.3)	6 (23.1)	20 (76.9)	0.036
6–10 years	68 (29.4)	28 (41.2)	40 (58.8)	
11–15 years	51 (22.1)	26 (51.0)	25 (49.0)	
16–20 years	40 (17.3)	21 (52.5)	19 (47.5)	
Above 20 years	46 (19.9)	27 (58.7)	19 (41.3)	
**Farm area (Ha)**	0.69 (0.03–2.0)	0.73 (0.08–2.00)	0.65 (0.03–1.82)	0.211
**Nos of pond/farm**				
**1–5**	218 (94.4)	97 (44.5)	121 (55.5)	0.006
**6–10**	12 (5.2)	10 (83.3)	2 (16.7)	
**11–15**	1 (0.4)	1 (100.0)	0 (0.0)	
**Source of water**				
Groundwater	119 (51.5)	50 (42.0)	69 (58.0)	0.06
River/canal	89 (38.5)	48 (53.9)	41 (46.1)	
Rain	9 (3.9)	4 (44.4)	5 (55.6)	
Groundwater + river/canal	9 (3.9)	6 (66.7)	3 (33.3)	
Rain + nearby farm	5 (2.2)	0 (0.0)	5 (100.0)	
**Species stocked**				
Carp species	15 (6.5)	4 (26.7)	11 (73.3)	<0.001
Carp + tilapia	66 (28.6)	37 (56.1)	29 (43.9)	
Carp + tilapia + Other freshwater species	88 (38.1)	34 (38.6)	54 (61.4)	
Carp + tilapia + freshwater species + brackishwater species	28 (12.1)	22 (78.6)	6 (21.4)	
Carp + other freshwater species	34 (14.7)	11 (32.4)	23 (67.6)	
**Fingerling sources**				
Hatchery	87 (37.7)	42 (48.3)	45 (51.7)	0.038
Nursery	27 (11.7)	10 (37.0)	17 (63.0)	
Hatchery + nursery	76 (32.9)	32 (42.1)	44 (57.9)	
Hatchery + incoming water	32 (13.9)	22 (68.8)	10 (31.3)	
Others	9 (3.9)	2 (22.2)	7 (77.8)	
**Stocking density**	109,351 (2059–1,728,635)	80,364 (2059–1,728,635)	134,803 (3756–1,445,168)	0.057
**Carp % of total stock**	45.6 (20–100)	49.4 (20–100)	42.2 (20–100)	0.049

**Table 3:** Association between biosecurity practices and disease incidence.

Variables	All Farms	Reported Diseases		
	n = 231	Yes (n = 108)	No (n = 123)	
	n (%) or Mean (Min, Max)	n (%) or Mean (95% CI)		*p*
**Biosecurity management during pond preparation**				
**Fallow period**				
No fallow period	23 (10.0)	5 (21.7)	18 (78.3)	0.069
One week	30 (13.0)	16 (53.3)	14 (46.7)	
Fortnight	50 (21.6)	25 (50.0)	25 (50.0)	
One month	56 (24.2)	31 (555.4)	25 (44.6)	
More than one month	72 (31.2)	31 (43.1)	41 (56.9)	
**Pond preparation**				
Bleaching + pond bottom drying + liming	10 (4.3)	0 (0.0)	10 (100)	<0.001
Pond bottom drying + ploughing + liming	25 (10.8)	6 (24.0)	19 (76.0)	
Pond bottom drying + liming	135 (58.4)	80 (59.3)	55 (40.7)	
Liming	58 (25.1)	21 (36.2)	37 (63.8)	
No measures	3 (1.3)	1 (33.3)	2 (66.7)	
**Biosecurity management followed during stocking**				
**Biosecurity practices at stocking**				
Fish transport water discarded off farm + fish disinfection + fish health inspection	33 (14.3)	12 (36.4)	21 (63.6)	0.010
Fish transport water discarded off farm + fish health inspection	70 (30.3)	44 (62.9)	26 (37.1)	
Fish disinfection + fish health inspection	12 (5.2)	6 (50.0)	6 (50.0)	
Fish transport water discarded off farm + fish disinfection	9 (3.9)	2 (22.2)	7(77.80	
Fish disinfection	17 (7.4)	9 (52.9)	8 (47.1)	
Fish health inspection	22 (9.5)	13 (59.1)	9 (40.9)	
Fish transport water discarded off farm	21 (9.1)	6 (28.6)	15 (71.4)	
No action taken	47 (20.3)	16 (34.0)	31 (66.0)	
**Introduction of new stock with old stock**				
Yes	157 (68)	63 (40.1)	94 (59.9)	0.005
No	74 (32)	45 (60.8)	29 (39.2)	
**On-farm general biosecurity management**				
**Farm perimeter fence**				
Yes	79 (34.2)	40 (50.6)	39 (49.4)	0.408
No	152 (65.8)	68 (44.7)	84 (55.3)	
**Restriction of entrance for general people**				
Yes	31 (13.4)	12 (38.7)	19 (61.3)	0.440
No	200 (86.6)	96 (48.0)	104 (52.0)	
**Restriction of entrance for domestic or other animals**				
Yes	54 (23.4)	15 (27.8)	39 (72.2)	0.002
No	177 (76.6)	93 (52.5)	84 (47.5)	
**Shared equipment with other farm/s**				
Yes	155 (67.1)	73 (47.1)	82 (52.9)	0.889
No	76 (32.9)	35 (46.1)	41 (53.9)	
**Hired harvester**				
Yes	223 (96.5)	103 (46.2)	120 (53.8)	0.479
No	8 (3.5)	5 (62.5)	3 (37.5)	
**Hired harvesting equipment**				
Yes	223 (96.5)	104 (46.6)	119 (53.4)	0.565
No	8 (3.5)	4 (50.0)	4 (50.0)	

**Table 4:** Results of univariable regression analyses for disease incidence (yes/no).

Variables	(OR)	95% CI	*p*-Value	AIC
**Region**				275.37
Barishal	1		<0.001	
Chittagong	0.0761	0.0097–0.5994		
Dhaka	1.75	0.6116–5.0074		
Khulna	5.95	2.5046–14.1350		
Mymensingh	5.6	1.8226–17.2057		
Jashore	3.5921	1.7085–7.5523		
**Primary occupation**				310.16
Agriculture	1		<0.001	
Aquaculture	5.7233	2.1627–15.1455		
Business	2.9167	0.9138–9.3090		
Job	1.9608	0.59–6.5168		
Others	3.3333	0.3837–28.9537		
**Farming experience**				314.97
1–5 years	1		0.003	
6–10 years	0.429	0.153–1.203		
11–15 years	0.288	0.099–0.837		
16–20 years	0.271	0.09–0.818		
Above 20 years	0.211	0.071–0.625		
**Nos of pond/farm**			<0.001	309.68
0–5	1			
6–10	0.2782	0.55–1.4088		
11–15	0	Undefine		
**Species stocked**				307.23
Carp species	1		<0.001	
Carp + tilapia	0.285	0.0822–0.9882		
Carp + tilapia + other freshwater species	0.5775	0.1701–1.9605		
Carp + tilapia + freshwater species + brackishwater species	0.0992	0.0231–0.4260		
Carp + other freshwater species	0.7603	0.1969–2.9366		
**Fingerling sources**				318.84
Hatchery	1		0.038	
Nursery	1.5867	0.6535–3.8526		
Hatchery + nursery	1.2833	0.6905–2.3852		
Hatchery + incoming water	0.4242	0.1799–1.0002		
Others	3.2667	0.6421–16.6192		
**Carp % of total stock**			0.049	317.55
0–35	1			
36–70	0.233	0.1281–0.4238		
71–100	0.576	0.2567–1.2927		
**Biosecurity measures during pond preparation**				299.8
**No measure**	1		<0.001	
**Liming**	0.881	0.0753–10.3063		
**Pond bottom drying + liming**	0.3438	0.0304–3.8849		
**Pond bottom drying + ploughing + liming**	1.5833	0.1212–20.6874		
**Bleaching + pond bottom drying + liming**	Undefined	Undefined		
**Biosecurity practices at stocking**				316.48
Fish transport water discarded off farm + fish disinfection + fish health inspection	1		0.01	
Fish transport water discarded off farm + fish health inspection	0.3377	0.1430–0.7973		
Fish disinfection + fish health inspection	0.5714	0.1503–2.1725		
Fish transport water discarded off farm + fish disinfection	2	0.3567–11.2154		
Fish disinfection	0.5079	0.1549–1.6654		
Fish health inspection	0.3956	0.1308–1.1968		
Fish transport water discarded off farm	1.4286	0.4377–4.6630		
No action taken	1.1071	0.4363–2.8094		
**Introduction of new stock with old stock**	0.4319	0.2454–0.7602	0.005	314.59
**Restriction of entrance for domestic or other animals**	0.3474	0.1787–0.6752	0.002	312.73

**Table 5:** Results of multivariable regression analyses for disease incidence (yes/no).

Multivariable, Final Model	Estimate	Std. Error	z Value	Pr (>|z|)	Significant Level ^1^	AIC
(Intercept)	−1.79 × 10^1^	1.12 × 10^3^	−0.016	0.987252		243.39
Region: Chittagong	2.18 × 10^0^	1.16 × 10^0^	1.872	0.061192		
Region: Dhaka	5.32 × 10^−3^	6.65 × 10^−1^	0.008	0.993615		
Region: Jashore	−1.74 × 10^0^	6.34 × 10^−1^	−2.747	0.006009	**	
Region: Khulna	−2.73 × 10^0^	7.01 × 10^−1^	−3.901	9.59E-05	***	
Region: Mymensingh	−1.88 × 10^0^	7.71 × 10^−1^	−2.437	0.014816	*	
Farming experience (yr.)	4.87 × 10^−2^	2.48 × 10^−2^	1.961	0.04988	*	
Stocking spp.: carp + other freshwater spp.	2.28 × 10^0^	1.01 × 10^0^	2.259	0.023856	*	
Stocking spp.: carp + tilapia	2.37 × 10^0^	9.47 × 10^−1^	2.505	0.012242	*	
Stocking spp.: carp + tilapia+ freshwater spp. + brackishwater spp.	4.58 × 10^0^	1.33 × 10^0^	3.453	0.000554	***	
Stocking spp: carp + tilapia + other freshwater spp.	8.63 × 10^1^	1.03 × 10^0^	0.845	0.397954		
Avg. percentage of carp stocked	2.85 × 10^−2^	1.18 × 10^−2^	2.413	0.015838	*	
Pond prep. biosecurity: liming	1.62 × 10^1^	1.12 × 10^3^	0.014	0.988446		
Pond prep. biosecurity: no action	1.50 × 10^1^	1.12 × 10^3^	0.013	0.989279		
Pond prep. biosecurity: drying + liming	1.72 × 10^1^	1.12 × 10^3^	0.015	0.98772		
Pond prep. biosecurity: drying + liming + plowing	1.62 × 10^1^	1.12 × 10^3^	0.014	0.98847		
Biosecurity at stocking: fish disinfection + fish health inspection	−9.73 × 10^−1^	9.84 × 10^−1^	−0.989	0.322713		
Biosecurity at stocking: fish health inspection	−9.50 × 10^−1^	8.57 × 10^−1^	−1.109	0.267501		
Biosecurity at stocking: transport water discarded off farm	−1.23 × 10^0^	8.70 × 10^−1^	−1.415	0.156963		
Biosecurity at stocking: transport water discarded off farm + fish disinfection	−2.57 × 10^0^	1.11 × 10^0^	−2.307	0.021028	*	
Biosecurity at stocking: transport water discarded off farm + fish disinfection + fish health inspection	−2.67 × 10^0^	8.63 × 10^−1^	−3.092	0.001987	**	
Biosecurity at stocking: transport water discarded off farm	−1.83 × 10^0^	7.80 × 10^−1^	−2.35	0.018794	*	
Biosecurity at stocking: no action	−2.07 × 10^0^	7.81 × 10^−1^	−2.65	0.008049	**	
Restriction of entrance for domestic or other animals	−3.78 × 10^−1^	4.30 × 10^−1^	−0.88	0.378658		

^1^ Significant level codes: 0 ‘***’ 0.001 ‘**’ 0.01 ‘*’ 0.05.

## Data Availability

The data collected for this study are available in the article.

## Supplementary Materials

The following supporting information can be downloaded at: https://www.mdpi.com/article/10.3390/ani14060966/s1, Health and Biosecurity problems in carp farming.
