# Supplementary material for: Factors Influencing Disease Dynamics in Small-Scale Carp Polyculture in Bangladesh
Source: Animals (Basel). 2024 Mar 20;14(6):966. doi: 10.3390/ani14060966 (PMC10967620; doi:10.3390/ani14060966)
Supplement: Supplementary file 1 [file animals-14-00966-s001.zip › animals-2892365-supplementary.pdf]

Health and Biosecurity problems in carp farming

1. Farmer details:

i. Farmer name:

ii. Mobile/email/contract details:

Mobile

email

contract details

iii. Farmer age:

iv. Gender:

- Male
- Female

v. Education:

- ◯ No education
- ◯ Primary (1-5)
- ◯ Higher Secondary (6-12)
- ◯ University (Bachelor/MS/more)

vi. Occupation:

Primary occupation:

Secondary occupation:

viii. Farming experiences (Years):

2. Farm details:

i. Farm name:

ii. Address of the farm:

Village

Union

Sub-districts/Upazila

District

ii. Total Farm area
In decimal

iv. Total water spread area
In decimal

v. Number of ponds in the farm:

vi. Farm type:

- ◯ Seasonal
- ◯ perennial

vii. Culture type:

- ◯ Monoculture
- ◯ Polyculture

viii. Source of water:

- [ ] Ground water
- [ ] River or canal
- [ ] Rain
- [ ] Nearby farm
- [ ] others

If others please specify

---

3. Stocking information:

---

i. Species:

---

a. Carp species:

- [ ] Rohu
- [ ] Catla
- [ ] Silvercarp
- [ ] grass carp
- [ ] Mrigala
- [ ] Others
- [ ] common carp
- [ ] bighead carp
- [ ] Others

---

i. Species:

---

b. Catfish:

☐ Pangus ☐ Walking catfish ☐ Stinging catfish ☐ Tengra ☐ Others

i. Species:

c. Other freshwater species:

☐ Tilapia ☐ Climbing perch ☐ Snakehead ☐ Silver barb ☐ Freshwater prawn
☐ Others

i. Species:

d. Brackish water species:

☐ Seabass ☐ mullet ☐ milkfish ☐ Shrimp ☐ Others

ii. Source of fingerling:

- [ ] hatchery
- [ ] nursery
- [ ] wild caught
- [ ] incoming water

iii. Stocking number (total):

____

iv. Average percentage of Carp stocked?

____

4. Disease's information (In carp species)

i. Have you experienced any unusual mortality/diseases in carp species during last production cycle?

- ◯ Yes
- ◯ No

ii. If yes, Month and year of onset mortality/diseases?

yyyy-mm-dd

iii. Nature of mortality/diseases?

- ◯ Sudden
- ◯ Gradual
- ◯ Occasional
- ◯ Multiple events

iv. Duration of mortality/diseases?

- ◯ Within a day
- ◯ Within a week
- ◯ Within a fortnight
- ◯ Within a month
- ◯ More than a month

v. Percentage of total mortality ?

---

vi. Age and size of fish during diseases/mortality?

Culture days during diseases/mortlaity

Weight of fish during diseases/mortality

---

vii. Major clinical sign observed by the farmer?

- [ ] Exophthalmia
- [ ] Shrinkage of eye
- [ ] Eye opacification
- [ ] Hemorrhages on skin and body surface
- [ ] Lesion
- [ ] Fin rot
- [ ] Tail rot
- [ ] Scale protrusion
- [ ] Abdominal distension
- [ ] Gill paleness
- [ ] Gill rot
- [ ] Others

If others, please specify

---

viii. Major behavioral changes

- ☐ Erratic swimming
- ☐ Appetite
- ☐ No schooling
- ☐ Others

If others, please specify

____

ix. How do you dispose the dead fish?

- ○ Collect and discard to nearby river or canal
- ○ Collect and buried on farm
- ○ Collect and buried off farm
- ○ Burnt
- ○ Fed to another animal on farm
- ○ Sale to the market
- ○ Fish did not remove from the pond
- ○ Others

If others, please specify

____

x. Any treatment used?

____

a) Chemical treatment?

- ○ Yes
- ○ No

If yes please specify

____

Is the treatment effective?

- ○ Yes
- ○ No

b) Antibiotic treatment?

- ○ Yes
- ○ No

If yes please specify

Is the treatment effective?

- ◯ Yes
- ◯ No

c) Probiotic treatment?

- ◯ Yes
- ◯ no

If yes please specify

Is the treatment effective?

- ◯ Yes
- ◯ No

d) Other treatment?

- ◯ Yes
- ◯ No

If yes please specify

Is the treatment effective?

- ◯ Yes
- ◯ No

xi. Do you send any sample for diseases diagnosis?

- ◯ Yes
- ◯ No

If yes what was the diagnosis report?

---

5. Biosecurity Practices

---

i. Biosecurity followed during pond preparation

---

a) Fallow period:

- ◯ No fallow period
- ◯ 1 week
- ◯ Fortnight
- ◯ Month
- ◯ More than a month

b) Pond bottom drying:

- ◯ Yes
- ◯ No

c) Ploughing:

- ◯ Yes
- ◯ No

d) Bleaching:

- ◯ Yes
- ◯ No

If yes dose of bleaching

Kg/Decimal

____

e) Liming:

- ◯ Yes
- ◯ No

If yes dose of liming

Kg/Decimal

____

f) Other chemical treatment:

- ◯ Yes
- ◯ No

If yes, please specify the name

____

ii. Biosecurity followed during stocking

a) Vehicle tyre bath at the entry:

- Yes
- No

b) Fish disinfection:

- Yes
- No

c) Fish transport water discarded off farm:

- Yes
- No

d) Fish health inspection/quarantine before stocking:

- Yes
- No

e) Introduction of new stock with old stock:

- Yes
- No

iii. Use of on farm biosecurity

---

a) Farm perimeter fence?

- Yes
- No

b) Restriction of entrance for general people?

- Yes
- No

c) Restriction of entrance for domestic or other animals?

- Yes
- No

d) Vehicle disinfection?

- Yes
- No

e) Footbath?

- Yes
- No

f) Hand disinfection?

- Yes
- No

g) Equipment disinfection?

- Yes
- No

h) Shared equipment with other farm/s?

- Yes
- No

i) Hired harvester?

- Yes
- No

j) Hired harvesting equipment?

- Yes
- No

k) Disinfection guideline for visitor?

- Yes
- No

iv. Biosecurity practices after diseases outbreak/mortality event

a) Follow complete harvesting:

- Yes
- No

b) Disinfect pond water:

- Yes
- No

If yes how? Please specify name and dose of treatment used

c) Continue production cycle without any disinfection:

- ◯ Yes
- ◯ No

e) Any observation of similar diseases occurrence again and again?

- ◯ Yes
- ◯ No

If yes how frequent

- ◯ 2 times a year
- ◯ 3 times a year
- ◯ 4 times a year
- ◯ last year and this year
- ◯ Continuing for last few years

Severity of the diseases during multiple events?

- ◯ More severe than earlier
- ◯ Less severe than earlier
- ◯ Similar with earlier

6. Farmer's observation and opinion

____

i. Any stressor factor for diseases occurrences? Please specify

____

ii. Any diseases occurrences in nearby farms and spread from that farm to his farm?

- ◯ Yes
- ◯ No

if yes was it similar types with the neighbor farm?

- ◯ Yes
- ◯ No

iii. What measures farmer take to prevent diseases occurrences? Please specify

____

iv. Did he received any training on biosecurity?

- ◯ Yes
- ◯ No

v. What support he needs to follow proper biosecurity for farming?

---

vi. Any other information he would like to provide:
